# Illinois State Medical Society

**Published:** 1859-12

**Authors:** 


### ILLINOIS STATE MEDICAL SOCIETY.

At the annual meeting of the Illinois State Medical Society, held in Decatur, on the first Tuesday in June last, the undersigned were appointed a Committee on Practical Medicine.

In order to fully accomplish the object of the appointment, we must take the liberty to ask the following questions of our medical friends throughout the State, and respectfully solicit answers to the same:

1. What have been the most prevailing diseases in your locality during the year? Give causes, symptoms, treatment, and rate of mortality.

2. Has any unusual epidemic prevailed in your section of country? If so, give character, treatment, etc.

3. Do the ordinary diseases of your region seem to undergo changes from year to year? If so, what are those changes, and what different treatment is necessary?

4. Has Typhoid Fever been prevalent in your section of country? Give your views of the pathology of Typhoid Fever, also, your treatment, etc.

5. Has Cholera Infantum prevailed with you during the year? Give cause, treatment, etc.

6. Has Diphtheria been prevalent with you? If so, give your views of the disease, treatment, etc.

7. Has Stomatitis Materna prevailed in your vicinity? Give suggestions as to causes and treatment.

8. Have you any improvements to suggest in the treatment of any disease?

9. Please give Topographical Description—so far as practicable—of your locality, together with any facts that may come under your notice, which will be interesting to the profession.

Address Christopher Goodbrake, M. D., Chairman of Committee, at Clinton, DeWitt county, Illinois, as early as March 1st, 1860.

CHR. GOODBRAKE,

F. B. HALLER,

D. W. YOUNG, } *Committee.*

---

On account of the general interest felt at this moment in the subject of poisoning, as well as the intrinsic importance of the subject, we transfer to our columns the following editorial notices of the Smethurst case, from the London *British and Foreign Medico-Chirurgical Review* and the London *Lancet*, both authoritative publications in England. We intend to return to the subject of the chemical analysis in the Jumpertz case, which, being at this moment on trial, is not perhaps a proper subject of comment at present.

